# Human Epidermal Growth Factor Receptor 2 Negative, Low, and Overexpression in Breast Cancer Patients: Study on Recurrence-Free and Overall Survival

**DOI:** 10.14740/wjon2780

**Published:** 2026-06-25

**Authors:** Widyanti Soewoto, Kristanto Yuli Yarso, Ida Bagus Budhi Surya Adnyana, Wibisono Wibisono, Alifia Ramadhani Herida, Arie Assakandari, Diesta Maylitadara, Aldona Akhira Susanto, Azzahra Fadhlila Aulia Nisa, Cornelius Steve

**Affiliations:** a Department of Surgery, Sebelas Maret University, Dr. Moewardi Hospital Surakarta, Central Java, Indonesia; b Medical Faculty, Sebelas Maret University, Dr. Moewardi Hospital Surakarta, Central Java, Indonesia

**Keywords:** Breast cancer, HER2 expression, HER2-low, Overall survival, Recurrence-free survival, Prognosis

## Abstract

**Background:**

Human epidermal growth factor receptor 2 (HER2) expression is a key prognostic marker in breast cancer. Recently, HER2-low breast cancer has emerged as a potentially distinct subgroup; however, its prognostic significance remains controversial, particularly in real-world clinical settings. This study aimed to evaluate recurrence-free and overall survival (OS) across HER2 expression categories: HER2-negative, HER2-low, and HER2-overexpressing.

**Methods:**

This observational analytic study used a retrospective cohort design conducted at Dr. Moewardi Hospital, a tertiary referral center in Central Java, Indonesia. A total of 2,310 breast cancer patients with complete HER2 immunohistochemistry (IHC) results and documented survival data from 2018 to 2020 were included. HER2 status was classified as HER2-negative (IHC 0), HER2-low (IHC 1+ or IHC 2+ without amplification), and HER2-overexpression (HER2-positive). The primary outcome was OS, defined as the time from diagnosis to death from any cause. Survival was analyzed using the Kaplan–Meier method, with comparisons by log-rank test and risk estimation using Cox proportional hazards regression.

**Results:**

Of the patients, 43.6% were HER2-negative, 21.3% HER2-low, and 35.2% HER2-overexpressing. Kaplan–Meier analysis showed significant differences in OS among groups (log-rank χ^2^ = 17.150; P < 0.001). HER2-negative patients had the best outcomes (mean OS 6.38 years; median unreached), followed by HER2-low (mean OS 5.60 years; median 4.00 years), and HER2-overexpression (mean OS 4.80 years; median 3.00 years). Significant differences were observed between HER2-negative vs. HER2-low (P = 0.015) and HER2-negative vs. HER2-overexpression (P < 0.001), but not between HER2-low and HER2-overexpression (P = 0.356). In Cox analysis, HER2-negative status reduced mortality risk by 22.2% compared with HER2-overexpression (hazard ratio 0.778; 95% confidence interval, 0.679–0.892; P < 0.001), while HER2-low showed no significant difference (hazard ratio 0.935; P = 0.413).

**Conclusions:**

HER2 expression is a significant prognostic factor in breast cancer. HER2-low patients have worse survival than HER2-negative patients and outcomes comparable to HER2-overexpression. These findings suggest that HER2-low breast cancer should not be considered low-risk and have important implications for prognostic stratification and treatment planning.

## Introduction

Breast cancer is the most frequently diagnosed malignancy in women and remains one of the leading causes of cancer-related mortality worldwide, despite recent declines in death rates [1–3]. More than 20,000 new breast cancer cases were diagnosed globally in 2020, and an estimated 266 million women died from breast cancer [4]. Nearly half of all breast cancer cases occur in Asia, where the overall burden continues to rise. As the region is home to about 60% of the world’s population, it also carries a higher mortality-to-incidence ratio compared with Western countries. Survival outcomes vary widely across Asia: while the 5-year survival rate exceeds 90% in Japan and South Korea, it remains around 66% in India. In Indonesia, breast cancer also represents a major health challenge, with approximately 68,858 new cases and more than 22,000 deaths reported in 2020 [5, 6].

Traditionally, breast cancer has been classified into distinct molecular subtypes according to the expression status of the estrogen receptor (ER) and human epidermal growth factor receptor 2 (HER2), namely ER-positive/HER2-negative, HER2-positive, and triple-negative breast cancer (TNBC) [7]. Current immunohistochemistry (IHC) profiling further refines these into luminal A, luminal B, HER2 overexpression, and TNBC. HER2 status is evaluated using IHC and *in situ* hybridization (ISH), with HER2-positive cancer defined as IHC 3+ or IHC 2+ with ISH amplification. HER2-positive cancer represents only about 15–20% of all breast cancer cases, while the majority fall into the HER2-low (IHC 2+ with ISH-negative or IHC 1+) and HER2-zero (IHC 0) categories [4, 8]. These subtypes vary in their associated risk factors, treatment approaches, prognosis, likelihood of recurrence, and overall clinical outcomes [9].

Emerging evidence has demonstrated that breast cancers classified as HER2-low represent a biologically distinct subgroup, exhibiting several clinicopathological and molecular features that partially overlap with those observed in HER2-positive cancer. Recent advances in therapeutic strategies, particularly the development of next-generation HER2-targeted antibody–drug conjugates, have shown promising clinical activity in this population, thereby challenging the traditional binary classification of HER2 status. These findings have shift paradigm in the understanding of HER2 expression as a continuous spectrum rather than a dichotomous variable [9, 10].

Despite substantial progress in systemic therapies and improvements in early detection, a significant proportion of patients diagnosed with early-stage HER2-negative breast cancer continue to develop locoregional or distant recurrence. Disease relapse remains a major clinical challenge, as it is frequently associated with limited treatment options, reduced overall survival (OS), and a marked decline in health-related quality of life. This underscores the persistent unmet need for improved risk stratification, predictive biomarkers, and more effective personalized therapeutic approaches to identify patients at high risk of recurrence and optimize long-term clinical outcomes [9, 10].

The prognostic significance of HER2-low status remains uncertain, with studies reporting conflicting results. Some research has suggested that HER2-low breast cancer is associated with either better or worse outcomes than HER2-zero cancer [11–13], while other investigations have found no meaningful differences in survival once hormone receptor (HR) status is taken into account [11, 14, 15].

A meta-analysis showed that HER2-low status functions as an independent prognostic factor, demonstrating improved disease-free survival (DFS) with a hazard ratio of 0.86 (95% confidence interval (CI): 0.79–0.92; P < 0.001) and improved OS with a hazard ratio of 0.90 (95% CI: 0.85–0.95; P < 0.001) in the total study population. When stratified by HR status, similar findings were seen in the HR-positive group, with DFS at hazard ratio 0.86 (95% CI: 0.80–0.93; P < 0.001) and OS at hazard ratio 0.94 (95% CI: 0.90–0.98; P = 0.003). In HR-negative early breast cancer, no significant difference was found for DFS (P = 0.155), although OS remained better in the HER2-low subgroup (hazard ratio 0.88; 95% CI: 0.82–0.95; P = 0.001). Among patients with metastatic breast cancer (mBC), HER2-low cancer was significantly associated with improved OS regardless of HR status (P = 0.008 in the overall cohort; P = 0.013 in the HR-positive subgroup; P < 0.001 in the HR-negative subgroup). However, progression-free survival (PFS) did not differ substantially between HER2-low and HER2-zero cases, either in the total population or when analyzed by HR status (P = 0.710 overall; P = 0.192 in HR-positive; P = 0.103 in HR-negative). Although the meta-analysis indicated modest improvements in OS and DFS for HR-positive early breast cancer and better OS in metastatic disease, these effects were small and inconsistent across HR categories. As a result, current evidence remains insufficient to clearly define the prognostic role of HER2-low status, highlighting the need for further research [11, 16]. Due to conflicting and various results of the studies, we conduct a study to evaluate the OS of breast cancer patients based on the classification of HER2 levels.

## Materials and Methods

### Study design and setting

This study employed an observational analytic design with a retrospective cohort approach. The retrospective design was chosen to enable evaluation of long-term outcomes using routinely collected clinical and follow-up data. The primary objective was to assess whether differences in HER2 expression were associated with variations in OS among patients diagnosed with breast cancer.

The study was conducted at a tertiary referral hospital that provides comprehensive oncologic care for patients from Central Java and surrounding regions. The study population consisted of all patients who underwent oncological evaluation at this institution between January 2018 and December 2020. During the 3-year study period, a total of 3,591 patients were registered in the Oncology Department.

This study was conducted in accordance with the ethical standards of the Institutional Review Board (Ethics Committee) of Dr. Moewardi Hospital, Surakarta, Indonesia (No. 1.423/VII/HREC/2025), and adhered to the principles of the Helsinki Declaration for research involving human subjects.

### Participants

**Figure 1:**
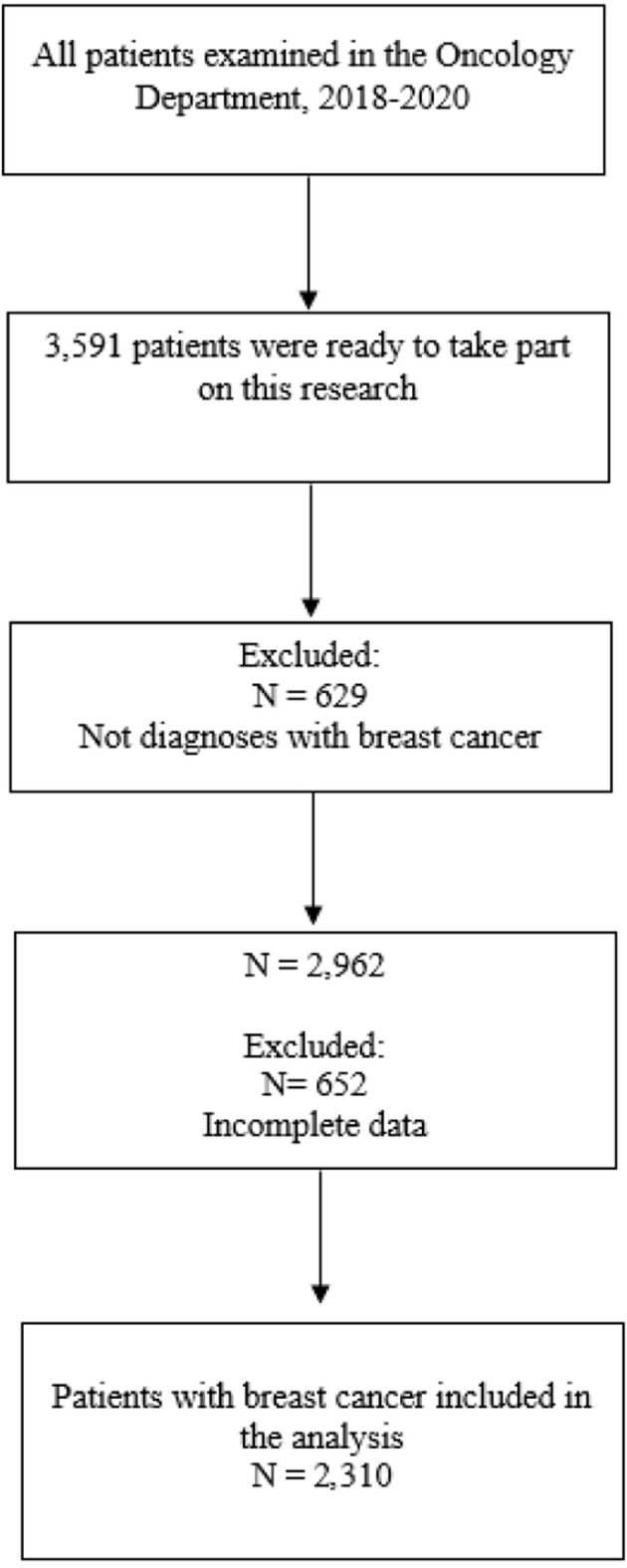
Flow chart of the study process.

A multistep screening process was applied to identify eligible patients as shown in Figure 1. From the initial 3,591 patients, 629 were excluded because they did not have a diagnosis of breast carcinoma. An additional 652 patients were excluded because of incomplete or missing essential clinical data, including unavailable HER2 IHC results, undocumented dates of diagnosis, or insufficient follow-up and survival information. Patients who met all predefined eligibility criteria after this screening process were subsequently included in the final analysis.

Inclusion criteria were: (1) histopathologically confirmed diagnosis of breast carcinoma; (2) availability of complete HER2 IHC results, classified as score 0, 1+, or 2+; (3) adequate documentation of survival time, including date of diagnosis and survival status; (4) patients with HER2 overexpression received anti-HER2 therapy, whereas patients with HER2-low and HER2-negative status did not receive anti-HER2 treatment.

Exclusion criteria included: (1) diagnoses other than breast carcinoma; (2) incomplete medical records or missing follow-up data; (3) absence of documented survival time, precluding inclusion in survival analysis.

### Statistical analysis

Statistical analyses were conducted using SPSS software version 26 (IBM Corp., Armonk, NY, USA). OS was estimated using the Kaplan–Meier method for each HER2 expression category. Survival differences between groups were assessed using the log-rank (Mantel–Cox) test.

Descriptive statistics were employed to summarize baseline demographic and clinicopathological characteristics of the study population. Continuous variables were reported as means with standard deviations for normally distributed data or as medians with appropriate measures of dispersion for non-normally distributed variables, as applicable. Categorical variables were presented as frequencies and percentages. All statistical tests were two-sided, and a P value of < 0.05 was considered to indicate statistical significance.

## Results

### Patients characteristics

A total of 2,310 patients were included in the final analysis. Baseline demographic and clinical characteristics are summarized in Table 1. More than half of the cohort was older than 55 years (51.2%; n = 1,184), followed by patients aged 45–55 years (35.5%; n = 821), while patients younger than 45 years comprised 13.2% (n = 305).

**Table 1:** Patients’ Characteristic

	Total study population	
	N	%
Age		
< 45	305	13.2
45–55	821	35.5
> 55	1,184	51.2
Stadium		
1	59	2.6
2	571	24.7
3	1,666	72.1
4	14	0.6
HER2 status		
Negative	1,007	43.6
Low	491	21.3
Overexpression	812	35.2
Outcome		
Death	1,450	62.7
Alive	860	37.2

HER2: human epidermal growth factor receptor 2.

Most patients presented with advanced-stage cancer. Stage III cancer was predominant (72.1%; n = 1,666), followed by stage II cancer (24.7%; n = 571). Early-stage (stage I) and metastatic (stage IV) cancer were uncommon, accounting for 2.6% (n = 59) and 0.6% (n = 14) of patients, respectively. Regarding HER2 expression, 43.6% (n = 1,007) of cancer were HER2-negative, 21.3% (n = 491) were classified as HER2-low, and 35.2% (n = 812) demonstrated HER2 overexpression. At the time of last follow-up, 1,450 patients (62.7%) had died, while 860 (37.2%) remained alive.

### OS analysis

**Figure 2:**
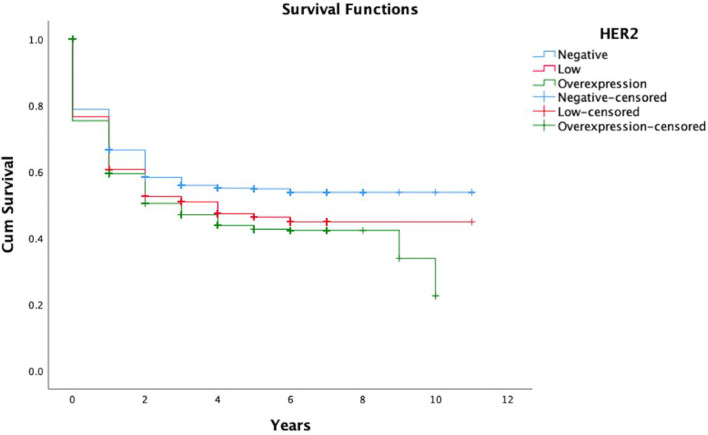
Kaplan–Meier survival probability according to HER2 status. The curves show the probability of overall survival among patients with HER2-negative (blue), HER2-low (red), and HER2-overexpression (green) breast cancer over an 11-year follow-up period. Tick marks indicate censored observations. HER2: human epidermal growth factor receptor 2.

OS was evaluated using the Kaplan–Meier method (Fig. 2) according to HER2 expression status: HER2-negative (group 0), HER2-low (group 1), and HER2-positive (group 2). Survival distributions differed significantly across groups (log-rank χ^2^ = 17.15; P < 0.001).

Patients with HER2-negative cancer demonstrated the most favorable outcomes, with a mean OS of 6.38 years (standard error (SE), 0.17; 95% CI, 6.05–6.71). Median OS was not reached during follow-up. In contrast, patients with HER2-low cancer had inferior survival, with a mean OS of 5.60 years (SE, 0.25; 95% CI, 5.10–6.09) and a median OS of 4.00 years (SE, 0.80; 95% CI, 2.43–5.57). The poorest outcomes were observed in patients with HER2-positive cancer, with a median OS of 3.00 years (95% CI, 2.26–3.74) and a mean OS of 4.80 years (95% CI, 4.44–5.17). The graphic illustration of the OS probability is shown in Figure 2 and data summary is shown in Table 2.

**Table 2:** Mean and Median Overall Survival According to HER2-Negative, HER2-Low, and HER2-Overexpression Status and Pairwise Log-Rank Comparisons of Overall Survival Between HER2-Negative, HER2-Low, and HER2-Overexpression Status

HER2	Mean				Median			
	Estimate	Standard error	95% Confidence interval		Estimate	Standard error	95% Confidence interval	
			Lower bound	Upper bound			Lower bound	Upper bound
Negative	6.382	0.169	6.050	6.713				
Low	5.585	0.251	5.092	6.078	4.000	0.803	2.426	5.574
Overexpression	4.793	0.187	4.427	5.160	3.000	0.372	2.271	3.729
Overall	6.134	0.140	5.858	6.409	3.000	0.383	2.249	3.751
			0			1		
			HER2	Chi-square	Sig.	Chi-square		Sig.
Log-rank (Mantel–Cox)			0			5.863		0.015
			1	5.863	0.015	0.852		0.356
			2	0.852	0.356			

HER2: human epidermal growth factor receptor 2.

*Post hoc* pairwise log-rank analyses demonstrated significant survival differences between HER2-negative and HER2-positive patients (χ^2^ = 15.81; P < 0.001) and between HER2-negative and HER2-low patients (χ^2^ = 5.86; P = 0.02). No statistically significant difference in OS was observed between HER2-low and HER2-positive patients (χ^2^ = 0.85; P = 0.36).

### Cox proportional hazards analysis

**Figure 3:**
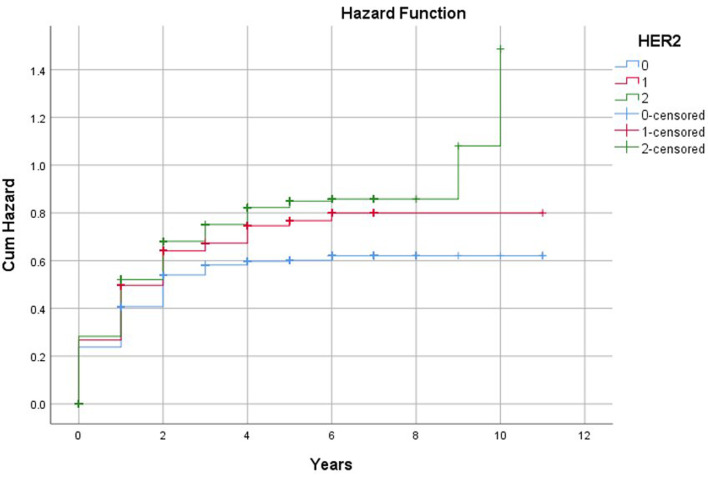
Cox proportional hazards regression analysis of overall survival based on HER2 expression. The curves illustrate the cumulative hazard of death among patients with HER2-negative (blue), HER2-low (red), and HER2-overexpression (green) breast cancer over the 11-year follow-up period. Higher values on the y-axis indicate a greater accumulated risk of mortality. HER2: human epidermal growth factor receptor 2.

In Cox proportional hazards regression analysis (Fig. 3), HER2 expression status was independently associated with mortality risk (model χ^2^ = 13.61; P = 0.001). Using HER2-positive cancer as the reference category, HER2-negative status was associated with a significantly reduced risk of death (hazard ratio, 0.78; 95% CI, 0.68–0.89; P < 0.001). In contrast, HER2-low status was not associated with a statistically significant difference in mortality risk compared to HER2-positive cancer (hazard ratio, 0.94; 95% CI, 0.80–1.10; P = 0.41).

Kaplan–Meier survival curves demonstrated a graded and persistent separation throughout follow-up, with HER2-negative patients exhibiting the highest survival probability, HER2-low patients showing intermediate outcomes, and HER2-positive patients experiencing the most rapid early decline in survival.

## Discussion

HER2 expression status was a significant determinant of OS in this study. Patients with HER2-negative cancer experienced the most favorable outcomes, with a mean OS of 6.38 years and a median survival that was not reached during follow-up. In contrast, HER2-positive cancer was associated with the poorest prognosis, with a median OS of 3.00 years. HER2-low cancer demonstrated intermediate numerical outcomes; however, survival among patients with HER2-low cancer was significantly inferior to that of HER2-negative patients and statistically indistinguishable from that of HER2-positive patients.

These findings indicate, within study population, HER2-low breast cancer represents a poor prognostic subgroup relative to HER2-negative cancer, while exhibiting clinical behavior comparable to HER2-positive cancer in terms of long-term survival. This observation is particularly relevant given the evolving recognition of HER2-low breast cancer as a biologically meaningful and therapeutically actionable entity following the demonstrated efficacy of novel antibody–drug conjugates, most notably trastuzumab deruxtecan [17]. Genomic and transcriptomic studies have further supported this paradigm by identifying molecular features that distinguish HER2-low cancer from HER2-zero cancer, indicating that HER2-low cancer may represent a biologically discrete subtype rather than a simple quantitative extension of HER2-negative breast cancer [18].

At the population level, evidence regarding the prognostic implications of HER2-low cancer has been inconsistent. Several meta-analyses and registry-based studies have suggested a modest survival advantage for HER2-low cancer compared with HER2-zero cancer. A meta-analysis including more than 670,000 patients reported a small but statistically significant improvement in OS for HER2-low breast cancer (hazard ratio, 0.90; 95% CI, 0.85–0.97) [3]. Similar findings have been observed in national cancer databases, particularly among patients with early-stage cancer [19, 20]. In addition, studies focusing on HR-positive breast cancer have reported improved DFS and OS associated with HER2-low status, especially among postmenopausal women [20, 21].

The prognostic significance of HER2-low breast cancer appears to vary according to cancer stage, biological subtype, and treatment context. In the metastatic setting, clinical outcomes have been heterogeneous, with inconsistent results across studies. Several retrospective and real-world analyses indicate that HER2-low status does not consistently confer either a favorable or unfavorable prognosis compared with HER2-zero cancer [22]. A large multicenter retrospective study of patients with metastatic breast cancer treated with endocrine therapy plus CDK4/6 inhibitors found no significant differences in PFS or OS between HER2-zero and HER2-low tumors, suggesting that low-level HER2 expression may not independently affect outcomes in this setting. The heterogeneity observed across studies may be driven by differences in patient populations, intrinsic molecular subtypes, prior treatment exposure, and dynamic changes in HER2 expression during disease progression. In addition, limitations of IHC assessment, including interobserver variability and discordance between primary and metastatic lesions, further complicate the interpretation of HER2-low status as a prognostic biomarker. Together, these factors highlight the complexity of defining the prognostic role of HER2-low cancer across different clinical settings [22].

In contrast, recent systematic review and meta-analysis reported significantly increased risk of progression or death among patients with HER2-low metastatic cancer treated with CDK4/6 inhibitors compared with those with HER2-zero cancer [23]. These discordant findings highlight the influence of cancer stage, molecular subtype, and treatment strategy on the prognostic impact of HER2-low status.

Consistent with this complexity, our findings indicate although patients with HER2-low breast cancer exhibited significantly poorer survival outcomes compared with those with HER2-negative cancer, their OS was statistically comparable to that observed in patients with HER2-positive cancer. This survival pattern suggests that, within our cohort, HER2-low breast cancer demonstrates clinical behavior more closely resembling HER2-positive cancer rather than HER2-negative. Importantly, these results do not support the notion of HER2-low status as a prognostically favorable intermediate subtype, but instead imply that low-level HER2 expression may be associated with a more aggressive disease phenotype in the absence of HER2-targeted therapy.

However, several mechanisms may account for these findings. Treatment heterogeneity and limited access to HER2-directed therapies in real-world clinical practice may allow oncogenic signaling driven by low-level HER2 expression to exert clinically meaningful effects, resulting in outcomes resembling those of HER2-positive cancer [17, 24]. In addition, molecular studies have demonstrated substantial biological overlap between HER2-low and HER2-positive cancer. In HR-positive cancer, HER2-low cancer has been shown to share metastatic dissemination patterns with HER2-positive cancer, including higher rates of lung and brain metastases and a greater likelihood of *de novo* stage IV presentation compared with HER2-zero cancer [18].

Dynamic changes in HER2 expression over the course of disease may further contribute to prognostic ambiguity [25, 26]. Several studies have documented discordance in HER2 status between primary cancer and metastatic lesions, with a subset of HER2-low cancer converting to HER2-positive cancer under therapeutic pressure [27, 28]. As a result, HER2 classification based on a single assessment at diagnosis may underestimate the biological aggressiveness of HER2-low cancer.

Moreover, the interaction between HER2-low status and HR expression appears to be a critical modifier of prognosis. Prior studies have suggested that any survival advantage associated with HER2-low cancer is largely confined to HR-positive cancer and may be absent in HR-negative cancer [19, 22, 23]. The absence of HR-stratified analyses in the present study may obscure clinically relevant subgroup-specific differences and contribute to the observed convergence in survival between HER2-low and HER2-positive cancer.

Taken together, these findings indicate that HER2-low breast cancer in our population does not provide clear survival advantage over HER2-positive cancer and should not be regarded as a low-risk disease. This conclusion is particularly relevant for clinical practice in settings where access to novel HER2-directed antibody–drug conjugates remains limited [23, 25]. In such contexts, patients with HER2-low cancer who do not receive targeted therapy may require surveillance and systemic treatment strategies more closely aligned with those HER2-positive cancer rather than HER2-negative cancer [9, 17, 26–29].

### Limitations

This study has several limitations. Its retrospective design introduces potential selection bias. In addition, detailed information regarding systemic therapies, including anti-HER2 agents, chemotherapy, and endocrine treatments, was not uniformly available, limiting treatment-adjusted survival analyses. The absence of HR-stratified analyses and the categorization of HER2 expression into three broad groups may have obscured underlying biological heterogeneity. Finally, sample size and follow-up duration may have limited the power to detect more subtle survival differences between subgroups.

### Future directions

Future studies should prioritize prospective, multicenter designs with long-term follow-up, comprehensive treatment data, HR stratification, and integrated molecular profiling [30–32]. Such approaches are essential to clarify the prognostic significance of HER2-low breast cancer and to determine whether it should be considered a distinct biological entity or primarily a quantitative extension of HER2-positive cancer in the era of precision oncology.

### Conclusion

This study demonstrates that HER2 expression status is a significant prognostic factor for OS in breast cancer, with a clear survival gradient observed across HER2-negative, HER2-low, and HER2-positive subgroups. Patients with HER2-negative cancer exhibited the most favorable survival outcomes, characterized by the longest mean OS and an unreached median survival time. In contrast, HER2-positive patients experienced the poorest prognosis, while the HER2-low group displayed an intermediate survival pattern. Importantly, despite this numerical gradient, no statistically significant difference in OS was observed between HER2-low and HER2-positive patients, a finding further supported by multivariate Cox regression analysis demonstrating comparable mortality risk between these two groups.

These findings indicate that, within our cohort and under prevailing real-world treatment conditions, HER2-low breast cancer does not confer a clear survival advantage over HER2-positive cancer, and may instead represent a biologically intermediate yet clinically aggressive phenotype. Although emerging molecular and therapeutic evidence supports HER2-low as a distinct biological subgroup, our results underscore that its prognostic implications remain highly context-dependent, being strongly influenced by treatment accessibility, HR status, and cancer stage.

From a clinical standpoint, these results emphasize that HER2-low breast cancer should not be underestimated as a low-risk disease, particularly in healthcare settings where access to HER2-directed therapies remains limited. As novel antibody–drug conjugates continue to reshape the therapeutic landscape for HER2-low cancer, future prospective studies with larger sample sizes, detailed treatment stratification, HR subgroup analyses, and integrated molecular profiling will be essential to refine risk stratification and to optimize personalized treatment strategies for this emerging breast cancer subtype.

## Data Availability

The data used in this study are confidential and are available solely for research purposes. Access to the data is restricted and may be granted only upon reasonable request, subject to approval by the relevant institutional authorities and in accordance with applicable ethical and privacy regulations.

## Abbreviations

CDK4/6: cyclin-dependent kinase 4 and 6
CI: confidence interval
χ^2^: (Chi-square)
DFS: disease-free survival
ER: estrogen receptor
HER2: human epidermal growth factor receptor 2
HER2-low: human epidermal growth factor receptor 2 low expression (IHC 1+ or IHC 2+ without amplification)
HER2-negative: human epidermal growth factor receptor 2 negative (IHC 0)
HER2-positive/HER2-overexpression: human epidermal growth factor receptor 2 overexpression (IHC 3+ or amplified)
HR: hormone receptor
IHC: immunohistochemistry
ISH: *in situ* hybridization
mBC: metastatic breast cancer
TNBC: triple-negative breast cancer
OS: overall survival
PFS: progression-free survival
SE: standard error
SPSS: Statistical Package for the Social Sciences
